# Duration of dry and humidified incubation of single-step embryo culture medium and oxygen tension during sham culture do not alter medium composition.

**DOI:** 10.12688/f1000research.109895.4

**Published:** 2022-05-30

**Authors:** Aswathi Cheredath, Shubhashree Uppangala, Gitanjali Asampille, Vani Lakshmi R., David Joseph, Keyur Raval, Nagana Gowda G. A., Guruprasad Kalthur, Satish Kumar Adiga

**Affiliations:** 1 Division of Clinical Embryology, Department of Reproductive Science, Kasturba Medical College, Manipal. Manipal Academy of Higher Education, Manipal, 576104, India; 2 Division of Reproductive Genetics, Department of Reproductive Science,, Kasturba Medical College, Manipal. Manipal Academy of Higher Education, Manipal, 576104, India; 3 Department of Data Science, Prasanna School of Public Health, Manipal Academy of Higher Education, Manipal, 576104, India; 4 NMR Research Centre, Indian Institute of Science, Bangalore, 576104, India; 5 Department of Chemical Engineering, National Institute of Technology Karnataka, Surathkal, India; 6 Northwest Metabolomics Research Center, Anesthesiology and Pain Medicine, University of Washington, Seattle, WA, USA; 7 Division of Reproductive Biology, Department of Reproductive Science, Kasturba Medical College, Manipal. Manipal Academy of Higher Education, Manipal, 576104, India

**Keywords:** Embryo metabolomics, Medium stability, Single step embryo culture, Sensitivity enhanced nuclear magnetic resonance spectroscopy

## Abstract

Background: The extended embryo culture using single-step medium gained popularity in clinical in vitro fertilisation (IVF). However, there are concerns about the degradation of unstable medium components and their negative effects on the developing embryos. Further, dry-incubation can increase osmolality, which can in-turn enhance the concentration of constituents of the media and their stability. Hence, this study was conducted to understand the immediate changes in the culture media constituents in relation to clinically comparable situations such as single-step extended embryo culture and use of dry and humidified-incubation in two-different gaseous conditions.

Methods: Commercially available single-step medium was sham-cultured in droplets under oil in two different conditions viz. dry (37°C; 6%CO 2; 5%O 2) and humidified (37°C; 6% CO 2; atmospheric O 2) for 0h, 72h, and 120h intervals. Droplets were subjected to the sensitivity-enhanced nuclear magnetic resonance (NMR)-based profiling using 800 MHz NMR equipped with a cryogenically cooled micro-coil (1.7mm) probe. NMR profile of the embryo culture medium between the two groups were comprehensively assessed.

Results: A total of ten amino acids and four energy substrates were identified from the culture medium. The medium constituents identified showed a non-significant increase in the dry-incubation group at 72h and then declined at 120h. Humidified incubation had no effects on the level of the identified medium constituents until 120h. No significant differences in the levels of medium constituents identified were observed between the dry and humidified-groups at various time-points tested.

Conclusions: A non-significant variation in the levels of medium constituents observed in the dry-incubation of single-step medium most unlikely to influence a clinical outcome. However, the impact of these subtle changes on the (epi)genetic integrity of the embryos in a clinical set-up to be addressed.

## Introduction

The embryo culture medium is expected to mimic an *in vivo* environment for the growth and health of the human preimplantation embryo *in vitro.* It has been shown that culture medium is one of the many crucial factors influencing the key process of fertilization and early embryogenesis ( Sunde *et al.,* 2016; Dumoulin *et al.,* 2010). On the other hand, culture medium can also affect the foetal growth and birthweight of the babies born through assisted reproductive technology (ART) ( Kleijkers *et al.,* 2016a; Nelissen *et al.,* 2012; Dumoulin *et al.,* 2010).

Several factors can impact the efficacy and stability of embryo culture media. These include the composition of the medium, osmolality, and conditions within the incubator such as humidity, gaseous state, pH, and temperature ( Mestres *et al.,* 2021; Tarahomi *et al., *2018, 2019; Swain *et al.,* 2016). Despite its importance, the exact formulation of commercially available embryo culture media is still unknown due to a lack of transparency in revealing the ingredients. However, choice of incubator and maintaining stable incubator conditions are laboratory-controlled factors that can strongly influence the medium’s stability.

Extended embryo culture in single step medium is gaining popularity due to its undisturbed culture, ability to monitor through time-lapse imaging, and importantly, the availability of single-step medium that supports embryonic development from one-cell to the blastocyst stage. However, one of the concerns with undisturbed extended embryo culture is the degradation of unstable components in the culture medium and their potential negative effects on the developing embryos. It has also been shown that uninterrupted embryo culture using single-step media in a dry atmosphere can increase osmolality, which can in turn enhance the concentration of constituents of the media and thereby alter the media’s stability ( Mestres *et al.,* 2021; Yumoto *et al.* 2019; Fawzy *et al.,* 2017).

Recently, a few studies tried to address the impact of factors influencing the stability of the embryo culture medium using various approaches ( Mestres *et al.,* 2021; Tarahomi *et al.,* 2018, 2019; Swain *et al.,* 2016). However, the availability of a large number of culture media and lack of uniformity in the culture methods employed by the embryologists, calls for extensive research on the individual products and methods used. In this study, experiments were specifically designed and executed to understand the immediate changes in the culture media constituents in relation to clinically comparable situations such as single-step extended embryo culture and use of dry and humidified incubation in two different gaseous conditions (dry incubation, 6% CO _2_, 5% O _2_; humidified incubation, 6% CO _2_; atmospheric O _2_). In order to understand the direct effects of these variables on the chemical composition of the medium, high-resolution 800 MHz nuclear magnetic resonance (NMR) spectroscopy with the help of 1.7 mm TX1 cryo-probe was used as the analytical tool to understand the composition of the culture medium.

## Methods

This prospective study was conducted at the Department of Clinical Embryology, Kasturba Medical College, Manipal and NMR Research Centre, Indian Institute of Science, Bangalore, India between September 2019-April 2021.

### Culture media

This study used a ready-to-use, protein supplemented V-ONESTEP medium (Cat No. V-OSM-20, Vitromed GmbH, Germany). Immediately upon arrival from the local distributor, ordered culture media were stored in a temperature monitored refrigerator (2–8°C). In total, three different batches were used in the study to investigate the media constituent changes. The measurements were taken before the expiry dates.

### Culture conditions

In order to mimic the conditions followed in the ART laboratory, the medium and dish preparation were handled in the same biosafety cabinet with the heat stage turned off. The medium in the bottle was taken out of the refrigerator, transferred to 14 mL Nunc tubes, and equilibrated in the humidified incubator (HeraCell 150i, Germany) at 37°C and 6% CO _2_ for 4 h.

**Figure 1.**
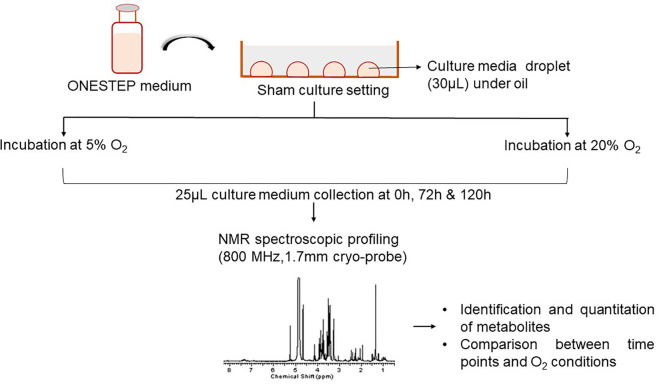
Overview of the experimental design. NMR=nuclear magnetic resonance.

As depicted in Figure 1, droplet culture on a petri dish was used in the study. Nine droplets of 30 μL equilibrated medium were placed on each petri dish (Falcon®, USA; Cat No. 353001), overlaid with 3 mL pre-incubated oil (Vitromed GmbH, Germany; Cat No V-OIL-P100). The dishes were placed inside the incubator immediately after the preparation and the time was noted (0 h). Two sham culture systems were used; i) dry incubation, 6% CO _2_, 5% O _2_ (MIRI® Multiroom incubator, ESCO Medical, Singapore); ii) humidified incubation, 6% CO _2_; atmospheric O _2_ (HeraCell 150i, Germany). Dry incubation contained moisture free environment whereas the humid atmosphere was attained by supplying the water (~2.5 litres) at the bottom of the incubator. The sham culture was performed in a stable condition for a period of 5 days. The media samples were collected in triplicates for analysis at three-time points from both groups, i.e. immediately after the preparation of the dish (0 h), on day 3 (72 h), and on day 5 (120 h).

From each droplet, 25 μL culture medium was carefully collected from randomly selected droplets without oil contamination and placed individually into labelled sterile cryovials, snap-frozen in liquid nitrogen, and then stored at -80°C until used for NMR analysis. To avoid oil contamination, only 25 μL of medium sample was aspirated from the 30 μL culture medium droplet that was placed in the sham culture dish. Further, pipette was inserted to the base of the culture dish after pressing the pipette plunger and once 25 μL was aspirated, pipette was dislodged from the base of the dish. Any slight oil contamination can be seen as separate layer or droplet within the medium since they are immiscible and such samples were not used in our study. A total of ten trials (N=10) were performed to confirm the reproducibility of the results.

### NMR sample preparation and analysis

The culture media samples were thawed for 10 minutes at room temperature. In total, 25 μL of each sample was diluted to 35 μL using deuterium oxide (D _2_O) solution containing a pre-calculated amount of TSP (Sodium salt of 2,2,3,3 tetradeutero 3-(trimethyl silyl) propionate) as a standard reference compound and transferred to 1.7 mm NMR tubes. Thus, all the constituents present in the culture media were diluted by a factor of 1.4. The dilution solvent was prepared by adding 0.05 g of TSP/mL D _2_O and diluting by a factor of 10 using D _2_O solvent. This solution (10 μL) was added to 25 μL culture medium sample to get a working solution containing 8.29 mM of TSP.

NMR experiments were performed on an 800 MHz Bruker AVANCE III NMR spectrometer equipped with a 1.7mm cryo-probe at 298 K. One dimensional (1D) ^1^H NMR spectra were obtained using the Carr-Purcell-Meiboom-Gill (CPMG) pulse sequence. CPMG 180-degree pulse train duration of 12 ms was used to suppress protein signals from the media. Each spectrum was obtained using 9615 Hz spectral width, 5 s relaxation delay, 16 k time-domain points, 4 dummy scans, and 256 transients. The time-domain data (FID's) were multiplied by a sine bell window function shifted by 90 ^o^ and zero-filled to 65536 points prior to Fourier transformation. Bruker Topspin version 3.6.2 software (RRID:SCR_014227) was used for NMR data acquisition and processing.

A total of 60 1D ^1^H spectra were acquired from ten trials. All data were analyzed using the Bruker TOPSPIN 3.6.2 software. Medium constituents were identified based on the literature and the characteristic peak integrals of the medium constituents were measured with respect to the TSP peak (which was normalized to 1.0). Subsequently, region wise (0.2 ppm) integration was performed using “intser” option. A total of 27 regions of the spectra obtained with peaks corresponding to the medium constituents were considered for the analysis.

### Statistical analysis

All the quantitative variables were represented as mean ± standard error of mean (SEM). Subsequently, a descriptive comparison of medium constituents across various gaseous and culture conditions were performed. Principal component (PC) analysis was carried out to explore the differences in the medium constituents across two culture conditions (dry and humidified) in two different gaseous conditions. A two-dimensional bi-plot ( Wickham, 2016) visualized the first two Principal Components (PCs; PC _1_ and PC _2_) that accounted for 99.41% of the variability in the data consisting of 27 integral regions captured across 60 samples from ten trials. The analysis was implemented in CRAN R 4.0 (RRID:SCR_003005).

Furthermore, the statistical significance for the sham culture across different time points and gaseous conditions was assessed using repeated-measures analysis of variance (ANOVA) in Jamovi 1.8.1 (RRID:SCR_016142). The level of significance was set at 5% throughout the study.

## Results

### 1D NMR analysis of V-ONESTEP medium

**Figure 2.**
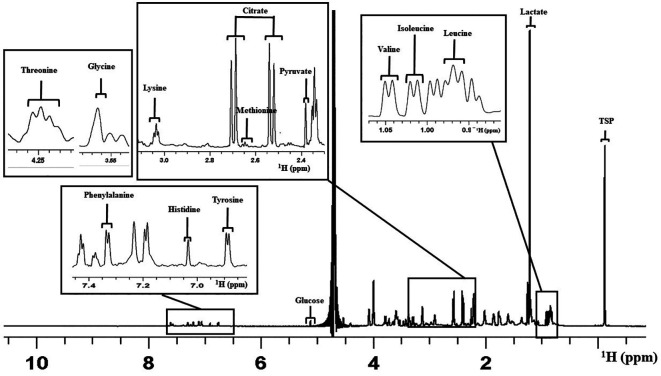
Representative figure for one dimensional ^1^H NMR spectrum of the ONESTEP embryo culture medium used in this study. The figure represents the assignment of peaks for different medium constituents. X-axis represents the chemical shift in parts per million (ppm). NMR=nuclear magnetic resonance.

Overall, 14 constituents of the culture medium were considered for the analysis as peaks that appeared were clear and distinct in all the spectra ( Cheredath *et al*., 2022). This included the amino acids such as Leucine (Leu), Isoleucine (Ile), Valine (Val), Methionine (Met), Glycine (Gly), Lysine (Lys), Threonine (Thr), Tyrosine (Tyr), Histidine (His), and Phenylalanine (Phe). Carbohydrate and other organic acid identified were Pyruvate (Pyr), Glucose (Glc), Lactate (Lac), and Citrate (Cit). Figure 2 shows a representative 1D proton NMR spectrum of V-ONESTEP medium with the assignments of peak.

### Effect of sham culture on the culture media constituents

*Time dependent changes in the level of medium constituents*

Dry incubation of V-ONESTEP medium at 5% O _2_ subjected to NMR analysis revealed no significant changes in the level of identified medium constituents at various time points tested. Interestingly, the level of all the identified medium constituents found to be increasing on day 3 (72 h) started declining thereafter. However, differences were not statistically significant for the trend observed ( Table 1). On the other hand, in the humidified incubation group, except pyruvate, the levels of all other medium constituents minimally altered on day 3 (72 h) and thereafter remained unchanged until day 5 (120 h). However, citrate and glycine showed a moderate non-significant variation on day 5 (120 h) ( Table 2).

**Table 1.** Sham incubation of one-step medium at 5% O _2_, subjected to nuclear magnetic resonance (NMR) analysis showing the relative concentration of medium constituents (normalized to TSP) at various time points.

Medium constituents identified	Relative concentration (Mean±SEM)			p value
	Baseline	Day 3	Day 5	
Leucine	1.97±0.14	2.38±0.28	2.19±0.26	0.60
Isoleucine	1.09±0.07	1.27±0.15	1.17±0.14	0.75
Valine	1.13±0.07	1.33±0.15	1.23±0.14	0.71
Lactate	23.98±1.57	28.38±3.29	26.20±3.10	0.65
Pyruvate	0.74±0.05	0.85±0.10	0.78±0.10	0.73
Citrate	3.48±0.23	3.96±0.45	3.80±0.45	0.87
Methionine	0.15±0.02	0.17±0.02	0.14±0.03	0.66
Lysine	0.78±0.05	0.85±0.10	0.80±0.09	0.87
Glycine	0.16±0.02	0.26±0.04	0.24±0.03	0.12
Threonine	0.35±0.04	0.47±0.06	0.43±0.06	0.13
Glucose	0.46±0.04	0.52±0.06	0.49±0.06	0.87
Tyrosine	0.36±0.02	0.42±0.05	0.39±0.05	0.77
Histidine	0.14±0.01	0.18±0.02	0.17±0.02	0.44
Phenylalanine	0.37±0.03	0.42±0.04	0.40±0.05	0.65

**Table 2.** Sham incubation of V-ONESTEP medium at 20% O _2_, subjected to nuclear magnetic resonance (NMR) analysis showing the relative concentration of medium constituents (normalized to TSP) at various time points. SEM=standard error of mean, TSP = Sodium salt of 2,2,3,3 tetradeutero 3-(trimethyl silyl) propionate.

Medium constituents identified	Relative concentration (Mean±SEM)			p value
	Baseline	Day 3	Day 5	
Leucine	1.97±0.14	2.10±0.16	2.09±0.25	0.99
Isoleucine	1.09±0.07	1.11±0.08	1.10±0.13	0.87
Valine	1.13±0.07	1.17±0.07	1.15±0.13	0.89
Lactate	23.98±1.57	25.43±1.82	25.18±3.09	0.99
Pyruvate	0.74±0.05	0.74±0.06	0.74±0.09	0.79
Citrate	3.48±0.23	3.61±0.24	3.48±0.42	0.81
Methionine	0.15±0.02	0.12±0.02	0.11±0.02	0.12
Lysine	0.78±0.05	0.75±0.04	0.76±0.09	0.48
Glycine	0.16±0.02	0.18±0.02	0.22±0.04	0.41
Threonine	0.35±0.04	0.42±0.05	0.43±0.06	0.28
Glucose	0.46±0.04	0.48±0.05	0.47±0.06	0.80
Tyrosine	0.36±0.02	0.38±0.03	0.37±0.04	0.93
Histidine	0.14±0.01	0.15±0.02	0.15±0.02	0.96
Phenyl alanine	0.37±0.03	0.39±0.03	0.39±0.05	0.99

*Impact of dry/5% O _2_ incubation vs humidified/atmospheric O _2_ incubation on the level of medium constituents*

**Figure 3.**
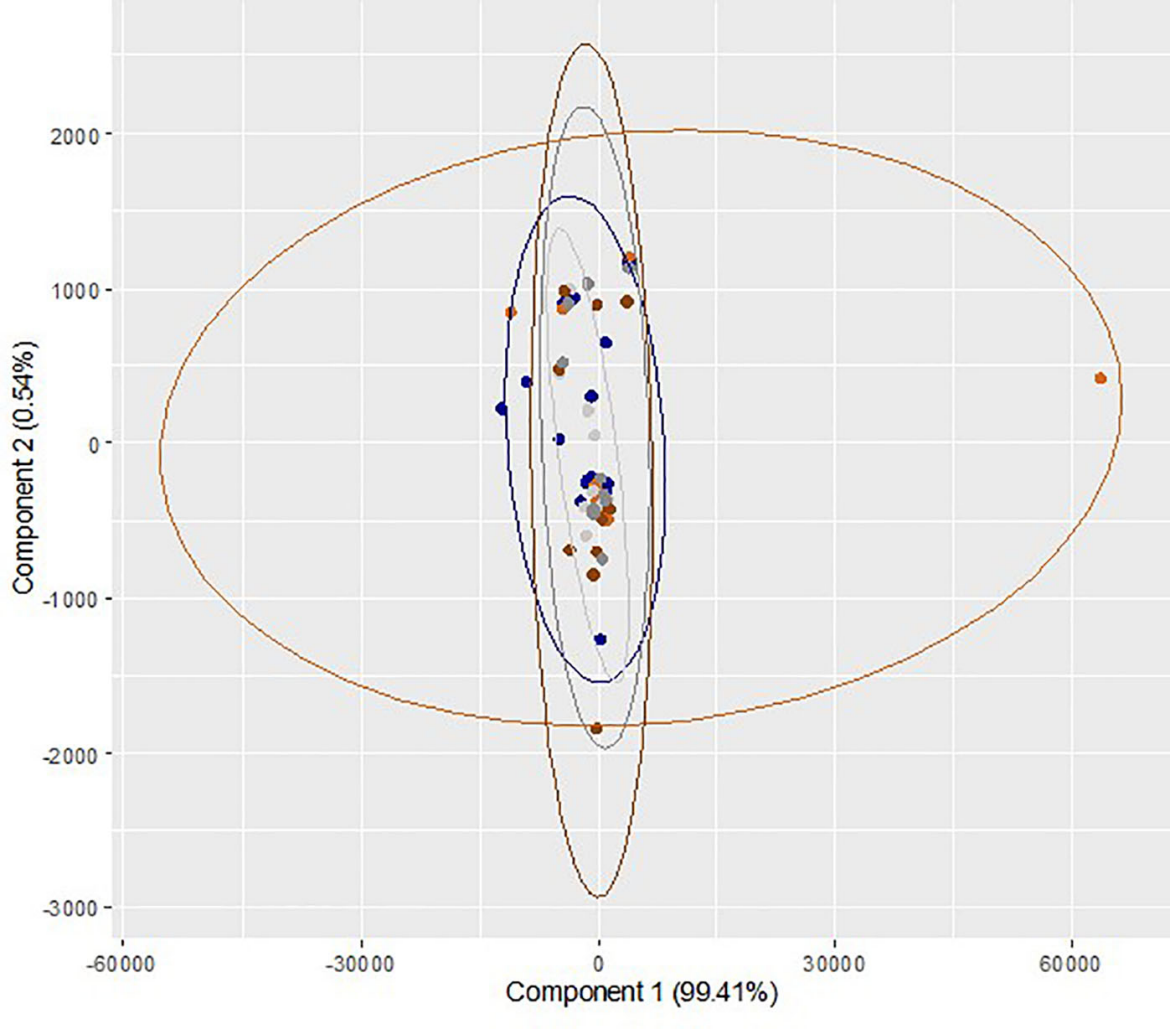
Principal component analysis (bi-plot) of the region wise integrals of sham culture performed at humidified/atmospheric oxygen and dry/physiologic oxygen level and collected at different time interval (0 h, 72 h and 120 h). Blue color ( 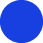) represents sample collected at 0h (baseline control) and light ash color ( 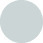) represents the sham culture performed at humidified/atmospheric O _2_ level and collected at 72 h, whereas orange color ( 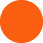) represents the sham culture performed at dry/physiological O _2_ level and collected at 72 h. Dark grey color ( 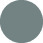) represents the sham culture performed at humidified/atmospheric O _2_ level and collected at 120 h and dark chocolate color ( 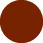) represents the sham culture performed at 5 dry/physiological O _2_ level and collected at 120 h.

Comparison of medium constituents between dry/5% O _2_ incubation and humidified/atmospheric O _2_ incubation at different time points is presented in Table 3. Though the level of all the identified medium constituents in dry/5% O _2_ incubation on day 3 and day 5 was higher than humidified/atmospheric O _2_ incubation group, the differences were not statistically significant. A multivariate exploration of the 27 integral regions captured from 60 samples from ten trials was performed using two-dimensional PC bi-plots (PC _1_ and PC _2_). The results of repeated measures ANOVA (Wilk's Lambda Method) reveal that there is no significant difference in the mean values of the relative concentration of medium constituents across the three time points (p *=*0.96) and two gaseous conditions (p *=*0.65). Also, there is no statistically significant interaction effect (p *=*0.69). These observations demonstrate no identifiable differentiation between the identified medium constituents of sham culture performed at different incubator conditions at different time points ( Figure 3). Overall, the effects of different incubator conditions and oxygen levels on sham culture of the ONESTEP media were not significant.

**Table 3.** Comparison of relative concentration of medium constituents (normalized to TSP) between dry/5% O _2_ incubation and humidified/atmospheric O _2_ incubation at different time points. SEM=standard error of mean, TSP = Sodium salt of 2,2,3,3 tetradeutero 3-(trimethyl silyl) propionate.

Medium constituents identified	Day 3			Day 5		
	Relative concentration (Mean±SEM)		p value	Relative concentration (Mean±SEM)		p value
	5% O _2_	Atmospheric O _2_		5% O _2_	Atmospheric O _2_	
Leucine	2.38±0.28	2.10±0.16	0.40	2.19±0.26	2.09±0.25	0.78
Isoleucine	1.27±0.15	1.11±0.08	0.35	1.17±0.14	1.10±0.10	0.74
Valine	1.33±0.15	1.17±0.07	0.37	1.23±0.14	1.15±0.13	0.69
Lactate	28.38±3.29	25.43±1.82	0.44	26.20±3.10	25.18±3.09	0.82
Pyruvate	0.85±0.10	0.74±0.06	0.34	0.78±0.10	0.74±0.09	0.79
Citrate	3.96±0.45	3.60±0.24	0.49	3.80±0.45	3.48±0.42	0.61
Methionine	0.17±0.02	0.12±0.02	0.05	0.14±0.03	0.11±0.02	0.46
Lysine	0.85±0.10	0.75±0.04	0.36	0.80±0.10	0.76±0.09	0.75
Glycine	0.26±0.04	0.18±0.02	0.11	0.24±0.03	0.22±0.04	0.70
Threonine	0.47±0.06	0.42±0.05	0.52	0.43±0.06	0.43±0.06	0.99
Glucose	0.52±0.06	0.48±0.05	0.60	0.49±0.06	0.47±0.06	0.87
Tyrosine	0.42±0.05	0.38±0.03	0.53	0.39±0.05	0.37±0.04	0.72
Histidine	0.18±0.02	0.15±0.02	0.41	0.17±0.02	0.15±0.02	0.44
Phenylalanine	0.42±0.04	0.39±0.03	0.62	0.40±0.05	0.39±0.05	0.86

## Discussion

The primary objective of this study was to test the stability of the single step culture medium and its interaction with the oxygen level (physiologic and atmospheric) within the dry and humidified incubation conditions. The end point assessment by analyzing the NMR signature of embryo culture medium at different time points revealed no significant extended culture impact using dry or humidified incubation at varying oxygen levels.

Several culture media are available commercially for human preimplantation embryo culture. Due to popularity, most of the available media are now designed to support uninterrupted extended culture until day 5 of development. However, one of the concerns with undisturbed extended embryo culture is the degradation of unstable components in the culture medium and their potential adverse effects on the developing embryos. It was found that ammonium is accumulated in the ready-to-use IVF culture media during incubation at 37°C ( Kleijkers *et al.,* 2016b), which may have a significant adverse effect on developing embryos. Despite its importance, manufacturers often do not disclose media composition and there is no clear evidence for the ideal formulation of the media used in ART ( Tarahomi *et al.,* 2019; Morbeck *et al.,* 2014a,b). Furthermore, there is no conclusive data comparing the stability of media when used in conjunction with non-humidified incubators and low oxygen culture system with humidified incubation at physiological oxygen level.

Earlier, Tarahomi *et al.,* (2019) analyzed the effects of storage and sham culture on 15 ready-to-use culture media and found that sham culture of the analysed media had a significant effect on the concentrations of 13 of the 37 analyzed components (Calcium, Phosphate, Albumin, total amount of Proteins, Tyrosine, Alanine, Methionine, Glycine, Leucine, Asparagine, Arginine, Proline, and Histidine). Though our study also had a similar objective, the use of a sensitivity-enhanced experiments using high frequency (800 MHz) NMR spectrometer facilitated the analysis of spent culture medium constituents with improved resolution and sensitivity. Further, the cryogenically cooled micro-coil probe (1.7 mm) provided an extreme boost (>10 fold) to sensitivity. The use of this CryoMicroProbe ^TM^ enabled fast NMR data acquisition with a more than 200-fold reduction in experiment time. Hence, we believe that this tool is extremely useful for investigating even subtle changes in the levels of medium constituents between the experimental groups tested in this study. It has been shown that medium components may vary depending on the time from production to use ( Tarahomi *et al.*, 2019). However, the study could not maintain the uniform time gap between the date of manufacture and usage as the manufacture date was not available. As per the manufacturer’s specifications, medium should be used within 7 days after opening the bottle which was strictly followed in our experiments.

The effect on the culturing pre-implantation human embryos at physiological oxygen level was considered beneficial as it mimics *in vivo* situation ( van Montfoot *et al.,* 2020; Kasterskin *et al.,* 2013; Meintjes *et al.,* 2009; Fischer and Bavister, 1993). However, a recent retrospective study conducted between 2011 and 2013 found that oxygen level during embryo culture does not affect the live birth rate, birth weight, and gestational age ( Castillo *et al.,* 2020). This study was limited by its retrospective nature and results were primarily based on sequential media. We believe that our experiments helped compare two commonly employed incubator conditions precisely by keeping other variables comparable. A multivariate exploration of the corresponding medium constituents’ integral regions captured across the samples using principal component analysis for the sham cultures across different oxygen levels and incubator types. This approach addressed the association between all the medium constituents present in the medium and not restricted to the fourteen constituents identified in this study.

Embryo culture incubator is one of the critical factors that determine the stability of the culture media a ( Simopoulou *et al.,* 2018). Conventional/standard incubator is humidified and provides approximately 20% oxygen level ( Castillo *et al.,* 2020). On the other hand, a dry incubator with a controlled oxygen level can change the osmolality of the culture medium possibly through evaporation ( Mestres *et al.,* 2021 Mullen, 2021). In addition, recent theoretical model has predicted that oil density and oil thickness above the embryo culture medium along with surface area to volume ratio can significantly contribute towards rise in osmolality of the medium in dry incubation conditions ( Mullen, 2021). However, the current study used constant volume of the oil (3 mL) throughout the study for overlay that can at least maintain the uniform oil thickness above the culture medium in all trials performed. In addition, oil from the same batch was used for both the sets of experiments. We have noticed an increase in the level of all the identified medium constituents during 72h dry incubation. However, the differences were not statistically significant. Interestingly no further increase in medium constituents was evident on day 5 (120h). Instead, there was a downward trend, which was again not statistically significant. At the moment, it is not possible to explain the reason behind this observation. In contrast, medium constituent levels did not change either on day 3 or day 5 in the humidified incubator.

The limitations of our study are i) use of only one commercially available singe step medium ii) not testing the osmolality of the medium, iii) excluding embryos in the culture and iv) not having humidified incubation group for low oxygen group as an incubator with this specification was not available in the present set up. Hence, it is not possible to confirm the exclusive impact of oxygen tension on the medium constituents.

## Conclusion

This study demonstrated only a non-significant variation in the medium constituents across the dry and humidified incubation systems using the NMR approach. Hence, the slight changes are unlikely to have any negative influence on embryological and clinical outcomes. Extensive studies are required to understand the impact of these subtle changes on the genetic and epigenetic integrity of the embryos in the clinical setup.

## Data availability

### Underlying data

Open Science Framework: Duration of dry and humidified incubation of single-step embryo culture medium and oxygen tension during sham culture do not alter metabolomics signature. https://doi.org/10.17605/OSF.IO/RCNZD ( Cheredath *et al*., 2022).

Data are available under the terms of the Creative Commons Zero “No rights reserved” data waiver (CC0 1.0 Public domain dedication).
