# Supplementary material for: Duration of dry and humidified incubation of single-step embryo culture medium and oxygen tension during sham culture do not alter medium composition
Source: F1000Res. 2022 May 30;11:242. Originally published 2022 Feb 28. [Version 4] doi: 10.12688/f1000research.109895.4 (PMC9214662; doi:10.12688/f1000research.109895.4)
Supplement: Peer review history

# Peer review: Reviewer response for version 4

**DOI:** 10.5256/f1000research.134426.r139369

**Published:** 2022-05-31

**Authors:** Borut Kovačič, Peter Slatinšek

**Affiliations:** 1 Department of Reproductive Medicine and Gynaecological Endocrinology, University Medical Centre Maribor, Maribor, Slovenia; 2 University Medical Centre Maribor, Maribor, Slovenia

The paper is now approved.

Is the work clearly and accurately presented and does it cite the current literature?

Yes

If applicable, is the statistical analysis and its interpretation appropriate?

Partly

Are all the source data underlying the results available to ensure full reproducibility?

Partly

Is the study design appropriate and is the work technically sound?

Yes

Are the conclusions drawn adequately supported by the results?

Partly

Are sufficient details of methods and analysis provided to allow replication by others?

Partly

Reviewer Expertise:

Reproductive medicine, assisted reproductive techniques, biology of reproductive cells and embryos, embryo culture conditions

We confirm that we have read this submission and believe that we have an appropriate level of expertise to confirm that it is of an acceptable scientific standard.

# Peer review: Reviewer response for version 3

**DOI:** 10.5256/f1000research.134280.r138650

**Published:** 2022-05-24

**Authors:** Borut Kovačič, Peter Slatinšek

**Affiliations:** 1 Department of Reproductive Medicine and Gynaecological Endocrinology, University Medical Centre Maribor, Maribor, Slovenia; 2 University Medical Centre Maribor, Maribor, Slovenia

The amendment should cover not only the title but also the abstract. Please correct. Then the paper is acceptable for indexing

Is the work clearly and accurately presented and does it cite the current literature?

Yes

If applicable, is the statistical analysis and its interpretation appropriate?

Partly

Are all the source data underlying the results available to ensure full reproducibility?

Partly

Is the study design appropriate and is the work technically sound?

Yes

Are the conclusions drawn adequately supported by the results?

Partly

Are sufficient details of methods and analysis provided to allow replication by others?

Partly

Reviewer Expertise:

Reproductive medicine, assisted reproductive techniques, biology of reproductive cells and embryos, embryo culture conditions

We confirm that we have read this submission and believe that we have an appropriate level of expertise to confirm that it is of an acceptable scientific standard, however we have significant reservations, as outlined above.

# Peer review: Reviewer response for version 2

**DOI:** 10.5256/f1000research.133843.r137717

**Published:** 2022-05-19

**Authors:** Borut Kovačič, Peter Slatinšek

**Affiliations:** 1 Department of Reproductive Medicine and Gynaecological Endocrinology, University Medical Centre Maribor, Maribor, Slovenia; 2 University Medical Centre Maribor, Maribor, Slovenia

The authors have followed most of the recommendations. They have probably forgotten our last comment, which should also be taken into account:

 "Minor point: Metabolites are the products of metabolism. It would be better to use the term 'medium constituents or components' instead of 'medium metabolites'. Please correct this throughout the text. The term 'metabolomic signature' should also be replaced by a more appropriate term".

Is the work clearly and accurately presented and does it cite the current literature?

Yes

If applicable, is the statistical analysis and its interpretation appropriate?

Partly

Are all the source data underlying the results available to ensure full reproducibility?

Partly

Is the study design appropriate and is the work technically sound?

Yes

Are the conclusions drawn adequately supported by the results?

Partly

Are sufficient details of methods and analysis provided to allow replication by others?

Partly

Reviewer Expertise:

Reproductive medicine, assisted reproductive techniques, biology of reproductive cells and embryos, embryo culture conditions

We confirm that we have read this submission and believe that we have an appropriate level of expertise to confirm that it is of an acceptable scientific standard, however we have significant reservations, as outlined above.

# Response

**Published:** 2022-05-20

**Authors:** Satish Adiga

**Affiliations:** Manipal Academy of Higher Education, India

We apologize for missing the most important suggestion by the reviewers in their previous version which is purely unintentional. We have modified the manuscript accordingly and shared with the Editorial office.

# Peer review: Reviewer response for version 1

**DOI:** 10.5256/f1000research.121449.r129570

**Published:** 2022-04-22

**Authors:** Borut Kovačič, Peter Slatinšek

**Affiliations:** 1 Department of Reproductive Medicine and Gynaecological Endocrinology, University Medical Centre Maribor, Maribor, Slovenia; 2 University Medical Centre Maribor, Maribor, Slovenia

- Is the work clearly and accurately presented and does it cite the current literature?
  The authors investigated the effect of dry and humid atmosphere and 5% and 20% oxygen concentrations in incubators on the changes of the composition of sham human embryo culture media. A high frequency (800 MHz) NMR spectrometer was used to analyse the changes in the concentrations of specific media components after 72 and 120 h incubation. The results did not show significant differences in the concentration of some media components after prolonged culturing at different incubation conditions. The work is well presented and relevant literature was cited.
- Is the study design appropriate and does the work have academic merit?
  The study is original and interesting for reproductive biologists, but some potential factors of methodological origin that could have influenced the results should be commented on, particularly in the discussion.
- Are sufficient details of methods and analysis provided to allow replication by others?
  It would be useful to explain what is meant by a dry and a humid atmosphere. Please describe how a humid atmosphere is achieved in the incubator.
  It is important to mention the role of the oil used to prevent evaporation of the culture media. It should be mentioned in the Discussion that differences in the amount of overlayed oil can affect the results (osmolality of the medium and stability of the medium components). Was this taken into consideration during the study? Control measurements of the osmolality of the medium would be useful, and the authors mention this shortcoming in the limitations of the study.
  In the literature it has also been shown that the components of a medium from the same batch can vary depending on the time from production to use. Have you taken this finding into account? If not, please mention the possibility of this effect in the discussion by using the relevant citation.
  Was triplicate sampling done from the same droplet or from different droplets / dishes?
  As oil contamination is one of the main problems in the analysis of culture media components, the sampling method must be described in detail and it must be explained how oil contamination has been prevented.
- If applicable, is the statistical analysis and its interpretation appropriate?
  The main finding of this research was described as: »The level of all the identified metabolites found to be increasing on day 3 (72 h) started declining thereafter in a dry atmospere group«. Such a result is unexpected and the authors must try to find possible explanations. If they cannot confirm them with the results of other published studies, they should be self-critical enough to try to find possible reasons arising from possible methodological variations. Some suggestions have been given above.
  By averaging the concentrations of the individual components of the medium, it was possible to mask the possible influence of unknown factors arising from technical details or from differently aged culture media of the same batch. Wouldn't it make sense to do a matched pairs sampling and relevant statistical analysis in such studies? It would be useful to present this option in the discussion and to provide a warning to the authors of future similar studies.
- Are all the source data underlying the results available to ensure full reproducibility?
  The tables shown contain all the relevant numerical data.
- Are the conclusions drawn adequately supported by the results?
  The conclusion should focus on the results obtained from the analysis rather than on possible clinical outcomes that were not the subject of the study. I suggest the following correction to the conclusion:
  No significant differences in the concentrations of the selected culture media components after 120 hours of incubation were observed between dry and humidified atmosphere, nor between 5% and 20% oxygen concentration in the incubators. Only a very mild trend of increasing concentrations after 72 hours and then decreasing after 120 hours in dry incubators was observed, which could also be attributed to certain methodological reasons described in the discussion.

**Minor point**

 Metabolites are the products of metabolism. It would be better to use the term 'medium constituents or components' instead of 'medium metabolites'. Please correct this throughout the text. The term 'metabolomic signature' should also be replaced by a more appropriate term.

Is the work clearly and accurately presented and does it cite the current literature?

Yes

If applicable, is the statistical analysis and its interpretation appropriate?

Partly

Are all the source data underlying the results available to ensure full reproducibility?

Partly

Is the study design appropriate and is the work technically sound?

Yes

Are the conclusions drawn adequately supported by the results?

Partly

Are sufficient details of methods and analysis provided to allow replication by others?

Partly

Reviewer Expertise:

Reproductive medicine, assisted reproductive techniques, biology of reproductive cells and embryos, embryo culture conditions

We confirm that we have read this submission and believe that we have an appropriate level of expertise to confirm that it is of an acceptable scientific standard, however we have significant reservations, as outlined above.

# Response

**Published:** 2022-05-07

**Authors:** Satish Adiga

**Affiliations:** Manipal Academy of Higher Education, India

We thank reviewer for his constructive comments.

*What is meant by a dry and a humid atmosphere? Please describe how a humid atmosphere is achieved in the incubator.*

 Dry incubation contained moisture free environment whereas the humid atmosphere was attained by supplying the water (~2.5 litres) at the bottom of the incubator. This information has been added in the methodology section of the revised manuscript.

*It is important to mention the role of the oil used to prevent evaporation of the culture media. It should be mentioned in the Discussion that differences in the amount of overlayed oil can affect the results (osmolality of the medium and stability of the medium components). Was this taken into consideration during the study? Control measurements of the osmolality of the medium would be useful, and the authors mention this shortcoming in the limitations of the study.*

 We do agree with the reviewer’s point of view. Oil overlay plays an important role in preventing the medium evaporation and maintaining the osmolality of the medium. In addition, a recent theoretical model has predicted that oil density and oil thickness above the embryo culture medium along with surface area to volume ratio can significantly contribute towards rise in osmolality of the medium during dry incubation conditions (Mullen *et al.*, 2021). We have taken precaution to maintain a constant volume of oil (3mL) throughout the study to overlay the medium. In addition, oil from the same batch was used for both the sets of experiments. We have now discussed these issues in the revised manuscript. However, the osmolality of the medium was not measured which is now stated as a limitation.

*In the literature it has also been shown that the components of a medium from the same batch can vary depending on the time from production to use. Have you taken this finding into account? If not, please mention the possibility of this effect in the discussion by using the relevant citation*

 Yes. We do agree that storage can have significant effect on the medium components as discussed in the manuscript (Tarahomi *et al*., 2019). However, study could not maintain the uniform time gap between the date of manufacture and usage as the manufacture date was not available. As per the manufacturer’s specifications, medium should be used within 7 days after opening the bottle which was strictly followed in our experiments. Since we did not find differences in the metabolites tested, this aspect was not elaborated in the discussion. However, new version has these points.

*Was triplicate sampling was done from the same droplet or from different droplets / dishes? *Triplicate sampling was done from the different droplets placed in a same dish during each trial.

*As oil contamination is one of the main problems in the analysis of culture media components, the sampling method must be described in detail and it must be explained how oil contamination has been prevented.*

 We do agree that the oil contamination is one of the main concerns which can interfere in the NMR analysis of culture media. To minimise the oil contamination, we have collected only 25µL of medium from the 30µL culture medium droplet from the culture dish. Further, pipette was inserted to the base of the culture dish after pressing the pipette plunger and once 25 µL was aspirated, pipette was dislodged from the base of the dish. Any slight oil contamination can be seen as separate layer or droplet within the medium since they are immiscible. Such samples were not used in our study. Oil contamination can cause difficulty in shimming the sample while performing NMR profiling which was not experienced by us during NMR profiling.

*If applicable, is the statistical analysis and its interpretation appropriate? The main finding of this research was described as: »The level of all the identified metabolites found to be increasing on day 3 (72 h) started declining thereafter in a dry atmosphere group«. Such a result is unexpected and the authors must try to find possible explanations. If they cannot confirm them with the results of other published studies, they should be self-critical enough to try to find possible reasons arising from possible methodological variations. Some suggestions have been given above. By averaging the concentrations of the individual components of the medium, it was possible to mask the possible influence of unknown factors arising from technical details or from differently aged culture media of the same batch. Wouldn't it make sense to do a matched pairs sampling and relevant statistical analysis in such studies? It would be useful to present this option in the discussion and to provide a warning to the authors of future similar studies.*

 The trend observed in our study is unique and unfortunately there is no evidence/published reports that can explain the possible reason for the trend. Although, we have taken the average of each metabolite, it was not possible to ignore the influence of external factors that affected our analysis as stated by the reviewer. The primary objective in this study was to test whether there are any statistically significant differences in the average values of the metabolite levels across three time points (0 hr, 72 hr and 120 hr) and two groups. Since the study included three time points, paired sample t-tests based approach is not appropriate. Whereas, the repeated-measures ANOVA approach used here is a natural extension to the paired samples approach to incorporate multiple time points across two or more groups.

# Peer review: Reviewer response for version 1

**DOI:** 10.5256/f1000research.121449.r126045

**Published:** 2022-03-18

**Authors:** Erode N. Prabhakaran

**Affiliations:** 1 Department of Organic Chemistry, Indian Institution of Science, Bangalore, Karnataka, India

Since extended embryo culture using single-step medium is now quite popularly applied in clinical in-vitro fertilization, it is important to test this commercially available single-step media for stability and for changes in metabolite profiles with variations in conditions. The authors have used dry and humidified-incubation in two different gaseous conditions to test the time-dependent variations in 10 amino acids and four energy substrate metabolite signals. They have chosen to analyze this variation through high-sensitive 1.7 mm cryoprobe in 800 MHz NMR spectral analyses.

 The experiments are well-designed with a single medium – the single-step embryo culture – which is commercially available but is extensively in use. Appropriate sham-cultures were used along with the sample cultures, a sufficient number of times, to validate the data for reliability and reproducibility. In general, they observe no significant differences in metabolite levels upon change of conditions from dry to humidified incubation over 72 h. Interestingly, they notice a decline of the metabolite levels at 120 h – although the extent of variations are non-significant. These results have prompted them to infer that both of these two conditions should be similar in influencing the clinical outcome. This and such studies are important and essential, since embryo cultures need to accurately mimic in vivo environments of preimplantation embryo, and since they strongly influence subsequent fetal health and growth. I commend this work and suggest that further studies in similar lines be done to explore the influence of other conditions on the efficiencies of these crucial culture media.

 As an advancement to such studies, the results may be compared between sets of media that have clinical records in influencing the growth of both healthy embryos and embryos with unhealthy aberrations.

Is the work clearly and accurately presented and does it cite the current literature?

Yes

If applicable, is the statistical analysis and its interpretation appropriate?

Yes

Are all the source data underlying the results available to ensure full reproducibility?

Yes

Is the study design appropriate and is the work technically sound?

Yes

Are the conclusions drawn adequately supported by the results?

Yes

Are sufficient details of methods and analysis provided to allow replication by others?

Yes

Reviewer Expertise:

Chemical Biology, Transcription engineering, Weak interactions in Proteins, Protein egineering.

I confirm that I have read this submission and believe that I have an appropriate level of expertise to confirm that it is of an acceptable scientific standard.
